# A Constraint-Driven Automated Framework for Optimizing Multi-Tool Fiducial Configurations in Surgical Navigation

**DOI:** 10.3390/bioengineering13070786

**Published:** 2026-07-08

**Authors:** Yuhui Wang, Chuanba Liu, Yifei Wang, Boyu Yang, Tao Sun

**Affiliations:** 1 Key Laboratory of Mechanism Theory and Equipment Design of Ministry of Education, Tianjin University, Tianjin 300350, China; yuhuiwang@tju.edu.cn (Y.W.); yifeiw@tju.edu.cn (Y.W.); 2024201341@tju.edu.cn (B.Y.); stao@tju.edu.cn (T.S.); 2 International Institute for Innovative Design and Intelligent Manufacturing, Tianjin University, Shaoxing 312000, China

**Keywords:** optical tracking tools, optimization design, surgical navigation accuracy, fiducial marker configuration

## Abstract

The accuracy of optical tracking tools is crucial for surgical navigation. While commercial tools are reliable, their proprietary design knowledge limits accessibility and adaptability for specialized clinical and research applications. This study introduces an open, reproducible optimization framework based on point-based rigid registration theory, defining a unified pose estimation deviation metric and deriving its analytical expression for both expectation and variance. The approach incorporates constraints for intra-group uniqueness and inter-group compatibility, using exhaustive configuration generation and geometric evaluation to rank designs by predicted accuracy. Numerical simulations confirmed the derived formula, with under 5% average prediction error for the expectation and strong agreement for the variance. Optimized four-fiducial tools were compared to commercial references via tip calibration, distance measurement, and registration tests. Most optimized tools (75%) achieved accuracy comparable to or modestly better than commercial tools (e.g., tip calibration 0.22 mm vs. 0.28 mm, distance measurement 0.18 mm vs. 0.20 mm, FRE 0.13 mm vs. 0.16 mm, TRE 0.48 mm vs. 0.51 mm). All four experiments showed a strong positive correlation between the theoretical metric and measured error (Pearson’s r>0.98, all $p<2.2\times 10^{-7}$; exact Spearman $p\leq 2.8\times 10^{-6}$). Beyond these numerical results, the primary contribution is a systematic, open design methodology that formalizes knowledge historically proprietary to commercial vendors, enabling researchers and engineers to generate high-precision custom tracking tools for diverse surgical navigation scenarios.

## 1. Introduction

One of the core functions of modern surgical navigation systems is real-time tracking of surgical instruments and anatomical targets in physical space, typically achieved via an optical tracking system (OTS) [1]. These systems rely on devices equipped with identifiable fiducial markers. Active systems typically use infrared LEDs, while passive systems adopt reflective spheres, checkerboards, or similar patterns. These markers enable geometric calibration, establishing a local coordinate frame with clearly defined fiducial points and the instrument tip position [2]. During tracking, the optical sensor measures the positions of the fiducial markers in physical space and aligns them with their calibrated counterparts, thereby computing the rigid transformation that maps the device’s pose to the global coordinate system [3]. As the navigation process requires continuous and accurate determination of the spatial relationship between the lesion and the instrument, localization error in these tracking devices becomes a critical factor affecting overall system precision [4].

Commercial navigation platforms typically offer several types of tracking devices, including the dynamic reference frame (DRF) and a tracked probe. The DRF, rigidly attached to the patient’s body, establishes the intraoperative coordinate frame and ensures spatial consistency during patient or equipment movement [5]. The probe maintains a fixed structure relative to the DRF and is used for acquiring fiducial points during image registration [6]. Despite their high accuracy, off-the-shelf tracking solutions often lack flexibility and are ill-suited for research scenarios requiring diverse or custom configurations [7]. In practice, new assemblies must usually be tailored to specific clinical or research needs. However, without strict adherence to geometric design principles—both for individual assemblies and for groups used concurrently—custom designs may exhibit diminished identifiability and robustness, ultimately reducing the stability and accuracy of the OTS [8,9].

Tracking accuracy is typically assessed using point-based rigid registration theory, with key metrics including fiducial localization error (FLE), fiducial registration error (FRE), and target registration error (TRE) [10]. However, existing metrics exhibit limitations when evaluating structures like the DRF: FRE can be misleading as a global accuracy measure [11], while TRE only applies to devices with well-defined target points, such as probe tips, and cannot capture pose estimation error for DRFs [12]. This highlights the need for a unified performance metric that facilitates optimal hardware design. In addition, clinical procedures often require the simultaneous use of multiple tracking assemblies. This imposes design constraints such as geometric uniqueness and distinct geometric features for unambiguous identification, further increasing the complexity of configuration generation.

Numerous studies have investigated the configuration design of optical tools, aiming to improve tracking performance through optimized fiducial geometry. For optical probe tools, many works have been carried out based on the point-based rigid registration theory proposed by Fitzpatrick et al. [13]. Jay B. West et al. [14] systematically analyzed the influence of the layout of the fiducial points on the tip positioning error and proposed several optimization design schemes; B. De Coninck et al. [15] conducted theoretical and empirical analysis on the six-fiducial probe structure; Fan et al. [16] designed a configuration suitable for different workspaces based on a three-dimensional image marking instrument; Stephen Thompson et al. [17] constructed a tracking error model suitable for laparoscopic cameras based on the assumption that the fiducial error is an independent random variable, effectively reducing the overall positioning error. Several studies have extended Fitzpatrick’s theory [18,19], seeking to relax its assumptions on error independence or adapt its evaluation framework to complex real-world scenarios. Beyond optical probes, other work has addressed DRF design optimization, proposing various methods for generating recognizable geometric configurations. For example, Pintaric et al. [11] used a random local search algorithm to construct 500 to 1500 non-coplanar marker arrangements; Gierlach et al. [20] further proposed a smaller “marker star” structure and simplified the data processing pipeline by enhancing marker distinguishability; Brown et al. [21] established an open source DRF library based on 3D printing technology and compared its performance with commercial tools.

Beyond incremental accuracy optimization, a more fundamental challenge motivates this work: the design knowledge underlying commercial tracking tools remains proprietary and inaccessible to the research community. Commercial DRFs, while highly reliable, are optimized for general-purpose use and cannot be easily adapted to specialized clinical or research requirements. When custom tool geometries are needed—for example, to accommodate sterilization constraints in specific surgical workflows, restricted workspaces in minimally invasive procedures (e.g., transnasal skull base surgery, percutaneous spine interventions), robotic end-effector integration, or procedure-specific anatomical accessibility—researchers and engineers must either adapt existing commercial tools (often suboptimally) or design new tools without systematic guidance. Furthermore, the open-source surgical navigation community lacks reproducible, customizable tool designs for developing and benchmarking new algorithms. The proposed framework addresses this gap by providing an open, theoretically grounded, and reproducible methodology for designing high-accuracy optical tracking tools with specified geometric constraints.

While the aforementioned work has achieved some progress, the following major limitations remain. First, whereas prior error models (e.g., Fitzpatrick et al. [13], West et al. [14], Thompson et al. [17]) focus primarily on probe tip tracking error (TRE), the proposed metric simultaneously quantifies both positional and orientational deviation for DRFs—a tool category for which TRE is undefined—thereby providing a unified accuracy measure applicable to both probes and DRFs. Second, existing DRF generation methods (e.g., Pintaric et al. [11], Brown et al. [21]) optimize primarily for geometric identifiability; the proposed framework additionally ranks and screens candidate configurations by theoretically predicted tracking accuracy, directly integrating accuracy optimization into the design loop. Third, while prior work addresses within-tool geometric uniqueness, the present framework is—to the best of our knowledge—the first to formalize and algorithmically enforce inter-tool compatibility constraints for multi-DRF surgical workflows. To address these gaps, this paper systematically analyzes the relationship between fiducial marker configuration and DRF accuracy based on point-based rigid registration theory, establishes an accuracy calculation model incorporating both expectation and variance, and proposes a tracking accuracy evaluation metric that can be uniformly applied to both probe and DRF. Furthermore, an algorithm is designed to generate tool configurations that satisfy both within-tool uniqueness and inter-group compatibility constraints, with parameter sensitivity characterized through analytical partial derivatives. The configuration accuracy is ranked and screened through theoretical analysis. Finally, experimental verification demonstrates the effectiveness and practicality of the proposed design method.

## 2. Methods

### 2.1. Overview

**Figure 1:**
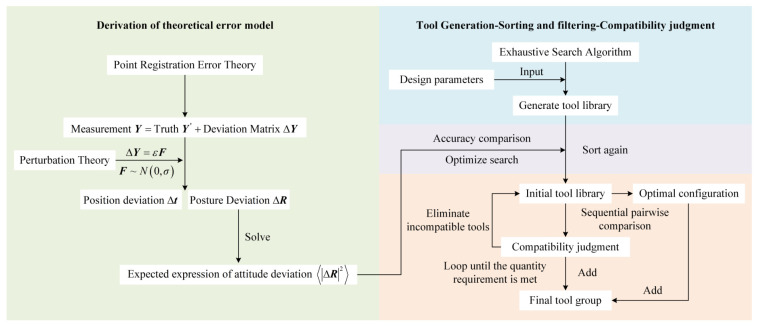
Wireframe diagram of the method overview. The first component constructs an error evaluation model and derives an analytical expression for the expected value of angular deviation. The second component develops a DRF configuration generation algorithm along with a compatibility determination algorithm for tool groups. The expected value of angular deviation obtained in the first component is subsequently applied to perform accuracy ranking and optimal design.

To address DRF evaluation and tool design optimization, this paper proposes a systematic approach to the optimal design of optical tracking tools. As illustrated in Figure 1, based on the point-based rigid registration theory, a novel accuracy evaluation metric is first proposed (Section 2.2). This metric overcomes the dependence of the TRE on specific target points by defining the pose estimation deviation (position deviation and orientation deviation) of the DRF coordinate system itself, thereby achieving a unified quantitative evaluation of the tracking accuracy of both the probe and the DRF. Subsequently, a design constraint-driven tool optimization framework is proposed based on this metric (Section 2.3). This framework integrates an exhaustive configuration library generation algorithm that satisfies both within-tool uniqueness constraints and cross-tool compatibility constraints. The proposed accuracy evaluation metric is then used to rank and optimize candidate designs within the library based on theoretical accuracy, ultimately generating a high-precision compatible tool set that meets practical application requirements.

### 2.2. Error Estimation Model

In practice, OTS devices unavoidably incur fiducial positioning errors due to measurement noise and environmental interference. These errors are inherently random and cannot be directly compensated, thus constituting a fundamental limitation of system performance. Given the limitations of existing metrics for assessing DRF accuracy, this section develops an error model for DRFs based on point-based rigid-registration theory and introduces a more generalizable accuracy evaluation metric.

**Figure 2:**
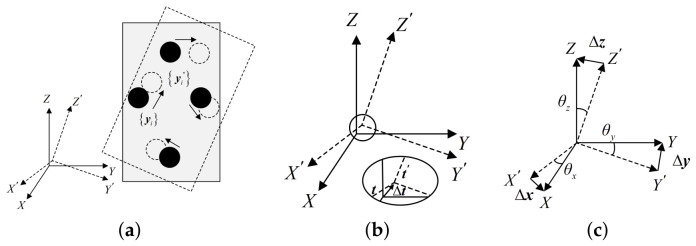
Schematic diagram of formula symbols. (**a**) Positioning error of the fiducial marker in the physical space coordinate system and the OTS. (**b**) The DRF true translation vector and measured translation vector. (**c**) The DRF true rotation matrix and the measured rotation matrix.

To evaluate DRF accuracy, both the tracking principle and error sources must be considered. After calibrating a DRF with *N* fiducial markers, the coordinates $x_{i}$ of each fiducial point in its local coordinate system {X} are obtained, forming a calibration matrix X. During tracking, the OTS measures the coordinates $y_{i}$ of each fiducial marker in the physical-space coordinate system {Y}, yielding a measurement matrix Y. By solving these matrices using the singular value decomposition (SVD) method [22], the rotation matrix R and translation vector t of the DRF in the spatial coordinate system {Y} at a certain moment can be obtained. However, as illustrated in Figure 2a, due to OTS positioning errors, a small deviation exists between the true coordinates of each fiducial point in the coordinate system {Y} and the measured coordinates, which can be expressed as

(1) $$Y=Y^{\prime }+\Delta Y$$

where the matrix $Y^{\prime }$ contains the real coordinates, and ΔY is the deviation matrix with small, unknown elements. Since the elements in the matrices $Y^{\prime }$ and ΔY are unknown, it is difficult to quantify the closeness between the true coordinate matrix and the measured coordinate matrix, so the error model needs to be simplified. The derivation relies on three standard assumptions from Fitzpatrick’s perturbation theory [13]: (1) the FLE is small relative to the inter-marker spacing (σ≪ fiducial spacing); (2) fiducial localization errors are independent and identically distributed (i.i.d.); and (3) the error distribution is isotropic and approximately Gaussian. Under these assumptions, when the FLE is much smaller than the fiducial point spacing (σ≪d, e.g., σ/d<0.005 for the ∼50–110 mm fiducial spacings used in this study), the influence of the overall rotation and translation on the error modeling can be ignored. The only difference between the two point sets is attributable to positioning error [23], that is, $Y^{\prime }=X$. For the deviation matrix ΔY, the fiducial positioning error in the OTS approximately follows a normal distribution within a certain range [24]. Therefore, it can be defined based on perturbation theory. Using the known solution and the power series of ε, the required solution ΔY=εF is written, where the elements in the matrix F are independent, identically distributed random variables that obey the normal distribution N(0,σ). Therefore, the true translation vector t of the DRF (Figure 2b) and the rotation matrix R (Figure 2c) can be calculated from the matrices X and Y.

Let the true translation vector be denoted by t and the measured translation vector by $t^{\prime }$. The position deviation $\Delta t=t-t^{\prime }$ is quantified by its squared Euclidean norm:

(2) $${\left|\Delta t\right|}^{2}={∥t-t^{\prime }∥}^{2}$$

The orientation deviation between the measured rotation matrix R and the true rotation matrix $R^{\prime }$ in space can be defined as

(3) $$\Delta R=R-R^{\prime }=\left[\Delta x\;\Delta y\;\Delta z\right]$$

The sum of the squares of all elements of the matrix ΔR is equal to the sum of the squares of the moduli of vectors Δx, Δy, and Δz. Equation (3) can also be expressed as

(4) $${\left|\Delta R\right|}^{2}=\sum_{i=1}^{3}\sum_{j=1}^{3}\Delta R_{ij}^{2}$$

Based on the above perturbation method, the approximate expressions of the orientation deviation ΔR and position deviation Δt can be derived. Because the definition of the point set coordinate system is arbitrary, the point set coordinate system can be redefined. For the convenience of calculation, the center of mass of the point set is selected as the origin of the coordinate system, that is, $\sum_{a=1}^{N}X_{ai}=0$. Under this condition, the actual position is $t_{i}^{\prime }=0$, so the approximate expression for the *i*-th component of the position deviation is $\Delta t_{i}=-t_{i}=-\frac{\varepsilon }{N}\sum_{a=1}^{N}F_{ai}$, where $F_{ai}$ represents the element of the *a*-th row and the *i*-th column of the matrix F. Therefore, the expected value of the squared position deviation norm ${\left|\Delta t\right|}^{2}=\sum_{i=1}^{3}{\left(\Delta t_{i}\right)}^{2}$ can be expressed as follows from Equation (2):

(5) $$\langle {\left|\Delta t\right|}^{2}\rangle =\langle t^{T}t\rangle =\frac{\varepsilon^{2}}{N^{2}}\sum_{i=1}^{3}\sum_{a=1}^{N}\sum_{b=1}^{N}\langle F_{ai}F_{bi}\rangle$$

Since the expectation of the product of two random variables is determined by their covariance, that is,

(6) E(xy)=cov(x,y)+E(x)E(y)

where *E* represents the expectation of the random variable, and cov is the covariance of the two random variables. Based on the assumption that the elements in the matrix F are independent and identically distributed, it can be seen that when a=b, the expectation of $F_{ai}F_{bi}$ is $\sigma^{2}$; when a≠b, the expectation of $F_{ai}F_{bi}$ is 0. Therefore, the expected expression of the position deviation Δt can be described as:

(7) $$\langle {\left|\Delta t\right|}^{2}\rangle =\langle t^{T}t\rangle =\frac{\varepsilon^{2}\sigma^{2}}{N^{2}}\sum_{i=1}^{3}\sum_{a=1}^{N}\sum_{b=1}^{N}\delta_{ab}=\frac{3\varepsilon^{2}\sigma^{2}}{N}$$

where $\delta_{ab}$ is the Kronecker delta function ($\delta_{ab}=0$ when a≠b; $\delta_{ab}=1$ when a=b). Equation (7) represents the mapping relationship between the expected value of the position deviation index and the tool design parameters. Equation (7) shows that the position deviation is related to the number of fiducial points and the fiducial positioning error. The greater the number of fiducial points and the smaller the fiducial positioning error, the smaller the position deviation.

For the characterization of the orientation deviation ΔR, since the elements of F are assumed to be identically distributed, the perturbation introduced in the error model is isotropic; consequently, the coordinate system orientation can be chosen arbitrarily. The direction chosen in this paper is the principal axis direction of the fiducial point distribution, that is, the true orientation $R^{\prime }=I$. Therefore, the orientation deviation ΔR=R−I. Note that ε=0 means that no error is introduced. At this time R=I, the rotation matrix R can be expanded using ε, which can be expressed as

(8) $$R-I=\varepsilon R^{\left(1\right)}+O\left(\varepsilon^{2}\right)$$

where $O(\varepsilon^{2})$ represents a second-order infinitesimal. From Equation (8), it can be concluded that for the second-order orientation deviation index, its expected value can be expressed as

(9) $$\langle {\left|\Delta R\right|}^{2}\rangle \approx \varepsilon^{2}\langle \sum_{i=1}^{3}\sum_{j=1}^{3}{R_{ij}^{\left(1\right)}}^{2}\rangle$$

At this point, the mapping relationship between the expected value of the orientation deviation index and the elements in the matrix $R^{\left(1\right)}$ is established. The matrix $R^{\left(1\right)}$ is obtained by expanding the matrix R. Combining the relevant properties of the rotation matrix R and the matrix singular value decomposition, the matrix $R^{\left(1\right)}$ is derived:

(10) $$R_{ij}^{\left(1\right)}=\frac{\Lambda_{ii}Q_{ij}-\Lambda_{jj}Q_{ji}}{\Lambda_{ii}^{2}+\Lambda_{jj}^{2}}$$

The subscript ij represents the element in the *i*-th row and *j*-th column of the matrix. The matrix $Q=U^{T}\hat{F}$, where the matrix U and the matrix Λ are the orthogonal matrix and diagonal matrix of the singular value decomposition of the matrix X. The elements in the matrix $\hat{F}$ are

(11) $${\hat{F}}_{aj}=F_{aj}-\frac{1}{N}\sum_{b=1}^{N}F_{bj}$$

Substituting Equation (10) into Equation (9) yields

(12) $$\langle {\left|\Delta R\right|}^{2}\rangle \approx \varepsilon^{2}\langle \sum_{i=1}^{3}\sum_{j=1}^{3}{R_{ij}^{\left(1\right)}}^{2}\rangle =\varepsilon^{2}\langle \sum_{i=1}^{3}\sum_{j=1}^{3}{\left(\frac{\Lambda_{ii}Q_{ij}-\Lambda_{jj}Q_{ji}}{\Lambda_{ii}^{2}+\Lambda_{jj}^{2}}\right)}^{2}\rangle$$

From the antisymmetry of the matrix $R^{\left(1\right)}$, we know that the diagonal elements of $R^{\left(1\right)}$ are 0, so Equation (12) can be changed to

(13) $$\langle {\left|\Delta R\right|}^{2}\rangle =\varepsilon^{2}\sum_{i=1}^{3}\sum_{j\neq i}^{3}\frac{\Lambda_{ii}\Lambda_{ii}\langle Q_{ij}Q_{ij}\rangle -2\Lambda_{ii}\Lambda_{jj}\langle Q_{ij}Q_{ji}\rangle +\Lambda_{jj}\Lambda_{jj}\langle Q_{ji}Q_{ji}\rangle }{\left(\Lambda_{ii}^{2}+\Lambda_{jj}^{2}\right)\left(\Lambda_{ii}^{2}+\Lambda_{jj}^{2}\right)}$$

The four terms in the numerator of Equation (13) are similar in form, all of which are $\Lambda_{\alpha \alpha }\Lambda_{\gamma \gamma }\langle Q_{\alpha \beta }Q_{\gamma \delta }\rangle$. According to Equation (11), the expected value of the elements in the matrix $\hat{F}$ can be obtained as

(14) $$\langle {\hat{F}}_{a\beta }{\hat{F}}_{b\delta }\rangle =\langle \left(F_{a\beta }-\frac{1}{N}\sum_{c=1}^{N}F_{c\beta }\right)\left(F_{b\delta }-\frac{1}{N}\sum_{d=1}^{N}F_{d\delta }\right)\rangle =\left(\delta_{ab}-\frac{1}{N}\right)\delta_{\beta \delta }\sigma^{2}$$

Therefore, its similarity can be simplified to

(15) $$\begin{array}{cc} \Lambda_{\alpha \alpha }\Lambda_{\gamma \gamma }\langle Q_{\alpha \beta }Q_{\gamma \delta }\rangle & =\delta_{\beta \delta }\sigma^{2}(\sum_{a=1}^{N}U_{a\alpha }U_{a\gamma }\Lambda_{\alpha \alpha }\Lambda_{\gamma \gamma }\\ \; & \;-\frac{1}{N}\sum_{a=1}^{N}U_{a\alpha }\Lambda_{\alpha \alpha }\sum_{b=1}^{N}U_{b\gamma }\Lambda_{\gamma \gamma })\\ \; & =\delta_{\alpha \gamma }\delta_{\beta \delta }\Lambda_{\alpha \alpha }^{2}\sigma^{2} \end{array}$$

Substituting it into Equation (13), we can get

(16) $$\langle {\left|\Delta R\right|}^{2}\rangle =\varepsilon^{2}\sigma^{2}\sum_{i=1}^{3}\sum_{j\neq i}^{3}\frac{1}{\left(\Lambda_{ii}^{2}+\Lambda_{jj}^{2}\right)}$$

Since the origin of the coordinate system is located at the center of mass of the point set, it can be seen that $\Lambda_{ii}^{2}+\Lambda_{jj}^{2}$ is the moment of inertia of the fiducial point distribution $M_{k}$ relative to the principal axis *k* (*i*, *j*, *k* represent the three principal axes of the coordinate system, namely the *x* axis, *y* axis, and *z* axis), which can be expressed as

(17) $$M_{k}=N\times f_{k}^{2}$$

where $f_{k}$ is the root mean square (RMS) distance from the fiducial point to the principal axis *k*, which can be calculated from the coordinates of the fiducial point *P*:

(18) $$f_{x}^{2}=\frac{1}{N}\sum_{i=1}^{N}x_{i}^{2},\;f_{y}^{2}=\frac{1}{N}\sum_{i=1}^{N}y_{i}^{2},\;f_{z}^{2}=\frac{1}{N}\left(\sum_{i=1}^{N}x_{i}^{2}+\sum_{i=1}^{N}y_{i}^{2}\right)$$

where $f_{z}$ denotes the RMS distance from each fiducial point to the *z*-axis (i.e., the in-plane spread for a planar configuration), which is non-zero even when all points are coplanar in the xy-plane.

According to the singular value decomposition property, the singular value Λ is directly related to the spatial distribution moment of inertia, and $\Lambda_{ii}^{2}$ corresponds to the variance of the point set in the direction of the principal axis, that is, $\Lambda_{ii}^{2}+\Lambda_{jj}^{2}=N\left(f_{i}^{2}+f_{j}^{2}\right)$. Substituting into Equation (16), the final expected value representation formula of the orientation deviation index is as follows:

(19) $$\langle {\left|\Delta R\right|}^{2}\rangle =\frac{\varepsilon^{2}\sigma^{2}}{N}\sum_{i=1}^{3}\sum_{j\neq i}^{3}\frac{1}{\left(f_{i}^{2}+f_{j}^{2}\right)}$$

Furthermore, the variance of the orientation deviation can be derived to characterize the dispersion of the tracking error.

(20) $${\left|\Delta R\right|}^{2}=\varepsilon^{2}{\left|R^{\left(1\right)}\right|}^{2}+O\left(\varepsilon^{3}\right)$$

and ${\left|\Delta R\right|}^{4}=\varepsilon^{4}{\left|R^{\left(1\right)}\right|}^{4}+O\left(\varepsilon^{5}\right)$, hence ${\mathrm{Var}(|\Delta R|}^{2})\approx \varepsilon^{4}\;\mathrm{Var}(|R^{\left(1\right)}{|}^{2})$.

From Equation (10) and the independence property of the matrix Q established in Equation (15), the off-diagonal elements $R_{ij}^{\left(1\right)}$ for i<j are independent Gaussian variables:

(21) $$R_{ij}^{\left(1\right)}∼N\;\left(0,\;\frac{\sigma^{2}}{\Lambda_{ii}^{2}+\Lambda_{jj}^{2}}\right),\;i<j$$

By the antisymmetry $R_{ij}^{\left(1\right)}=-R_{ji}^{\left(1\right)}$, the squared Frobenius norm $|R^{\left(1\right)}{|}^{2}=2\sum_{i<j}{\left(R_{ij}^{\left(1\right)}\right)}^{2}$ is a weighted sum of three independent $\chi^{2}\left(1\right)$ variables. Since $\mathrm{Var}(\chi^{2}\left(1\right))=2$,

(22) $$\begin{array}{cc} \mathrm{Var}(|R^{\left(1\right)}{|}^{2}) & =4\sigma^{4}\sum_{i<j}\frac{2}{{\left(\Lambda_{ii}^{2}+\Lambda_{jj}^{2}\right)}^{2}}\\ \; & =8\sigma^{4}\sum_{i<j}\frac{1}{{\left(\Lambda_{ii}^{2}+\Lambda_{jj}^{2}\right)}^{2}} \end{array}$$

Substituting $\Lambda_{ii}^{2}+\Lambda_{jj}^{2}=N\left(f_{i}^{2}+f_{j}^{2}\right)$ from Equation (17) yields the final variance expression:

(23) $$\mathrm{Var}({\left|\Delta R\right|}^{2})\approx \frac{8\varepsilon^{4}\sigma^{4}}{N^{2}}\sum_{i<j}\frac{1}{{\left(f_{i}^{2}+f_{j}^{2}\right)}^{2}}$$

Equivalently, $\mathrm{Var}({\left|\Delta R\right|}^{2})\approx \frac{4\varepsilon^{4}\sigma^{4}}{N^{2}}\sum_{i=1}^{3}\sum_{j\neq i}^{3}\frac{1}{{\left(f_{i}^{2}+f_{j}^{2}\right)}^{2}}$, and the standard deviation follows as

(24) $$\mathrm{SD}({\left|\Delta R\right|}^{2})\approx \frac{2\sqrt{2}\;\varepsilon^{2}\sigma^{2}}{N}\sqrt{\sum_{i<j}\frac{1}{{\left(f_{i}^{2}+f_{j}^{2}\right)}^{2}}}$$

The coefficient of variation (CV), obtained from Equations (19) and (23), is independent of ε and σ and depends only on the fiducial geometry:

(25) $${\mathrm{CV}}^{2}=2\cdot \frac{\sum_{i<j}{\left(f_{i}^{2}+f_{j}^{2}\right)}^{-2}}{{\left(\sum_{i<j}{\left(f_{i}^{2}+f_{j}^{2}\right)}^{-1}\right)}^{2}}$$

For an isotropic fiducial distribution ($f_{1}=f_{2}=f_{3}=f$), $\mathrm{CV}=\sqrt{2/3}\approx 0.816$. Both the expectation and variance decrease with the number of fiducial markers *N*, but the variance decreases faster ($\propto 1/N^{2}$ vs. ∝1/N), indicating that adding markers not only reduces the expected error but also substantially narrows its dispersion.

**Parameter Sensitivity Analysis.** To quantify how design parameters influence tracking accuracy, the partial derivatives of the expected orientation deviation (Equation (19)) with respect to key parameters are derived.

*Sensitivity to fiducial count N.* From Equation (19),

(26) $$\frac{\partial \langle |\Delta R{|}^{2}\rangle }{\partial N}=-\frac{\varepsilon^{2}\sigma^{2}}{N^{2}}\sum_{i=1}^{3}\sum_{j\neq i}^{3}\frac{1}{f_{i}^{2}+f_{j}^{2}}<0$$

The negative sign confirms that increasing *N* always improves accuracy. The $1/N^{2}$ dependence indicates diminishing returns: the marginal benefit of adding the *N*-th marker is proportional to $1/N^{2}$. For the case N=4 chosen in this study, doubling to N=8 would reduce $\langle |\Delta R{|}^{2}\rangle$ by a factor of 2 (from the 1/N term) while increasing the number of line segments from S=6 to S=28—a nearly five-fold increase in computational complexity for the configuration generation algorithm.

*Sensitivity to fiducial spread $f_{k}$.* For the RMS distance along principal axis *k*,

(27) $$\frac{\partial \langle |\Delta R{|}^{2}\rangle }{\partial f_{k}}=-\frac{2\varepsilon^{2}\sigma^{2}}{N}\sum_{j\neq k}\frac{f_{k}}{{\left(f_{k}^{2}+f_{j}^{2}\right)}^{2}}<0$$

The sensitivity scales as $f_{k}^{-3}$ for configurations where $f_{k}\gg f_{j}$, indicating a strong dependence on the smallest spatial extent. This provides theoretical justification for maximizing the fiducial spread within the workspace constraints.

*Sensitivity to fiducial localization error σ.*

(28) $$\frac{\partial \langle |\Delta R{|}^{2}\rangle }{\partial \sigma }=\frac{2\varepsilon^{2}\sigma }{N}\sum_{i=1}^{3}\sum_{j\neq i}^{3}\frac{1}{f_{i}^{2}+f_{j}^{2}}>0$$

The linear dependence on σ confirms that the metric is directly proportional to FLE variance, consistent with the linear error propagation expected from perturbation theory.

Together, these sensitivity relationships provide an analytical basis for selecting design parameters: (i) the fiducial count *N* should balance accuracy gains against computational and manufacturing complexity; (ii) the fiducial spread should be maximized within the physical workspace; and (iii) any reduction in FLE (e.g., through improved marker materials or calibration) yields proportional accuracy improvements across all configurations.

### 2.3. Tool Generation and Optimization Design

**Figure 3:**
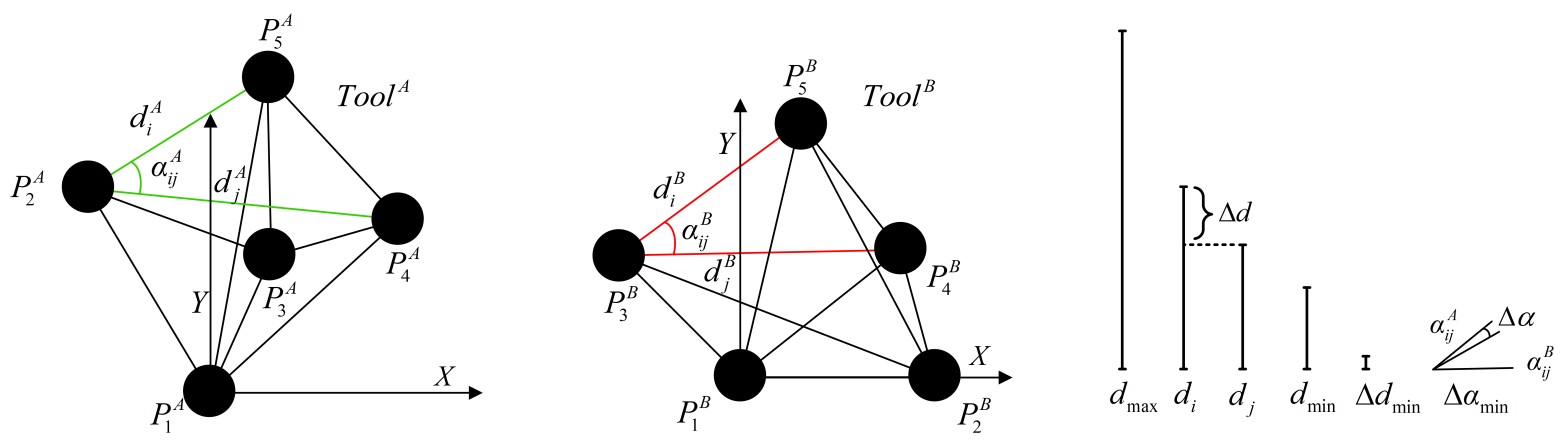
DRF within-tool and inter-group compatibility constraints and associated design parameters. Green lines: edge lengths $d_{i}$ of the reference DRF; red lines: edge lengths of the comparison DRF. Within a DRF group, the distance between fiducial points is denoted as $d_{i}$; the minimum distance as $d_{\min}$; the maximum distance as $d_{\max}$; and the minimum difference between distances as $\Delta d_{\min}$; the angle between two similar edges $d_{i}^{A}$ and $d_{j}^{B}$, as well as between two adjacent edges $d_{i}$ and $d_{j}$ in different groups, is denoted as $\alpha_{ij}$. The minimum angular difference between two adjacent angles of a similar edge $d_{i}^{A}$ and two adjacent angles of $d_{j}^{B}$ is denoted as $\alpha_{\min}$.

In practice, an OTS relies on distance and angular information between fiducial points for geometric matching and pose recognition. Therefore, to enable simultaneous identification of multiple tools with similar configurations, essential geometric constraints must be incorporated at the DRF configuration design stage. This study focuses on planar DRF configurations, which represent the most common design in current clinical practice due to manufacturing simplicity and favorable line-of-sight characteristics. The methodological framework—accuracy evaluation, constraint-driven generation, and compatibility screening—is general and can be extended to non-coplanar configurations (see Discussion). Figure 3 presents a schematic diagram illustrating the within-tool and inter-group configuration design parameter constraints. For an individual DRF, its geometric configuration must be unique to ensure stable recognition. Accordingly, the configuration design must satisfy the following within-tool constraints: (1) all edge lengths must be distinct; (2) all edge lengths must meet the minimum length $d_{\min}$, maximum segment length $d_{\max}$, and minimum difference $\Delta d_{\min}$ required by the system. These constraints prevent fiducial image occlusion, enhance system recognition capability, and ensure that the DRF has a reasonable physical size to meet clinical requirements.

In designing multiple configuration combinations, in addition to satisfying the uniqueness constraint of an individual DRF, inter-group compatibility constraints must be incorporated to ensure that the system can accurately distinguish the poses of multiple DRFs in space simultaneously. If two configurations share a set of line segments with similar lengths (i.e., a length difference less than $\Delta d_{\min}$), the corresponding angles must be further examined to determine whether they are sufficiently distinct. Specifically, a pair of DRFs is considered compatible only if the minimum angle difference between these corresponding angles exceeds a system-defined threshold $\alpha_{\min}$. This design strategy effectively prevents mirrored or geometrically similar configurations from coexisting within the DRF library, thereby enhancing the robustness of multi-tool collaborative tracking.

To achieve an exhaustive configuration design for a planar DRF with multiple fiducials, this study proposes a search method, as illustrated in Algorithm 1. The algorithm takes as input the number of fiducial points *N*, the fiducial edge length range $\left[d_{\min},\;d_{\max}\right]$, the minimum edge length difference $\Delta d_{\min}$, and the iteration step size *s*, and generates all valid line segment combinations that satisfy the within-tool constraints. Since the configuration of *N* fiducial points requires determining K=N(N−1)/2 edge lengths, and each pair of points has three degrees of freedom (position and rotation), fixing the global coordinate system necessitates subtracting three degrees of freedom. Therefore, at least 2N−3 edge lengths are required to fix the position. All remaining distances, U=K−2N+3, can be computed using triangulation, the cosine law, or iterative methods. The algorithm traverses the sequence by gradually increasing length, computes the remaining edge lengths using triangulation or the cosine law, and ranks each candidate configuration by accuracy based on the expected value of the pose deviation (Equation (19)). Finally, duplicate designs are removed to construct a candidate library C of configurations that satisfy the accuracy ranking criteria.

**Algorithm 1:** Size exhaustive generation and theoretical accuracy sorting algorithm

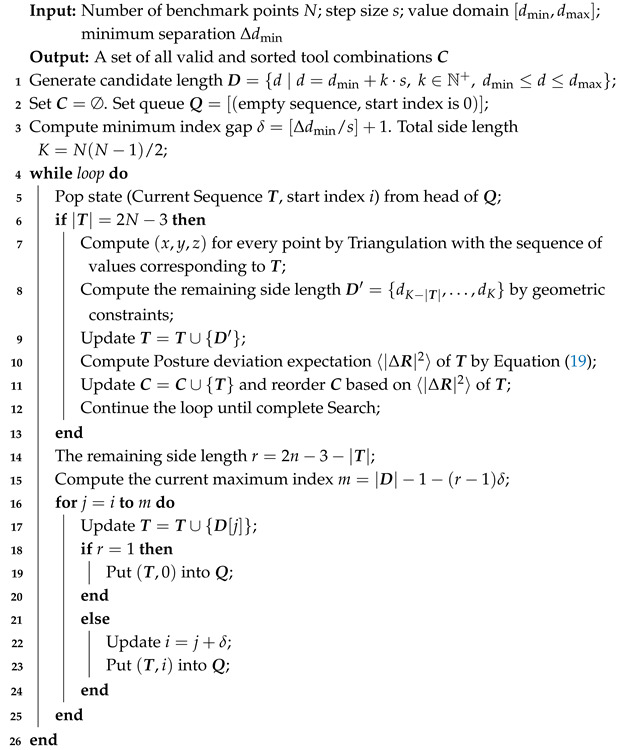



To select a group of compatible, high-precision tools from the candidate configuration library, this study develops a tool compatibility determination mechanism, as illustrated in Algorithm 2. The algorithm compares all edge-length pairs between candidate tools to determine whether any pair has similar edge lengths (i.e., a length difference less than $\Delta d_{\min}$). If two or more similar edge lengths exist, the corresponding angles are computed, and it is then determined whether the angle difference exceeds a threshold $\alpha_{\min}$. Only when the angle differences of all similar edges meet the requirements is the pair of tools considered compatible, after which a compatibility matrix M is constructed based on this criterion. By combining the above two algorithms, the tool with the best theoretical accuracy (i.e., the smallest $\langle |\Delta R{|}^{2}\rangle$) is selected from the candidate configuration library C as the seed tool $T_{0}$. Algorithm 2 is then used to screen all tools compatible with $T_{0}$, sort them by accuracy, and select the preferred tools for inclusion in the compatibility group. The process is iterated until the required library size is obtained.

**Algorithm 2:** Tool group compatibility judgment algorithm

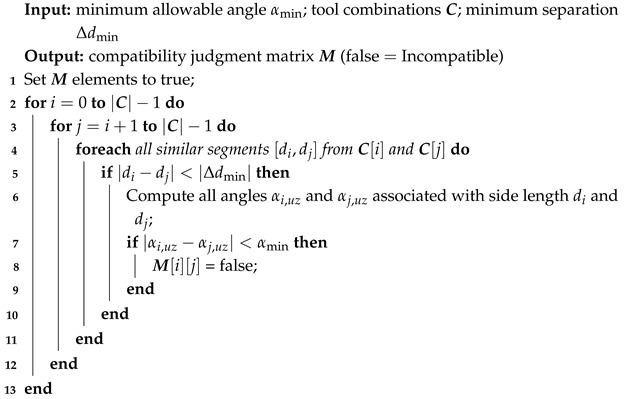



The discrimination thresholds $\Delta d_{\min}$ and $\alpha_{\min}$ play a critical role in the compatibility determination. Although these thresholds are set empirically in the current implementation, their order of magnitude can be justified through a semi-theoretical analysis relating them to OTS measurement uncertainty. Under the Gaussian FLE assumption, the distance measurement uncertainty between two independently localized fiducial markers is $\sigma_{d}=\sqrt{2}\;\sigma_{\mathrm{FLE}}$. For the Northern Digital Inc. (NDI) Polaris system, passive tracking trueness has been reported as 0.17±0.09 mm [25]; we adopt a conservative FLE estimate of approximately 0.25 mm, yielding $\sigma_{d}\approx 0.35$ mm. To ensure reliable discrimination at >99.7% confidence (the 3σ rule), the minimum edge length difference should satisfy $\Delta d_{\min}\geq 3\sigma_{d}\approx 1.06$ mm. The empirical choice of $\Delta d_{\min}=5$ mm in this study provides a conservative safety margin that accounts for additional uncertainty sources such as partial occlusion, varying marker visibility, and manufacturing tolerances. The angular threshold $\alpha_{\min}$ is geometrically coupled to $\Delta d_{\min}$: for two edges of length *d* differing by $\Delta d_{\min}$, the smallest resolvable angle is approximately $\alpha_{\min}\approx \arcsin\left(\Delta d_{\min}/d\right)$. With $d_{\min}=52$ mm and $\Delta d_{\min}=5$ mm, this yields $\alpha_{\min}\approx 5.5^{\circ }$; the more conservative empirical choice of $2^{\circ }$ reflects the ability of the OTS to resolve angular differences finer than the geometric bound when multiple edge-length constraints are simultaneously active. A fully rigorous derivation of optimal thresholds via a formal confusion-probability analysis, as well as the extension to non-Gaussian noise models, is left for future work.

## 3. Experiment

To verify the practical utility of the DRF configuration design and the effectiveness of the accuracy ranking method, this study conducted four types of verification experiments. Because the true pose of the DRF during use is unknown, direct measurement of its accuracy is not feasible; indirect measurement experiments were therefore designed according to the functions performed by the DRF in surgical navigation. Numerical simulation experiments validate the theoretical accuracy of the orientation deviation formula; tip calibration experiments assess probe tip calibration accuracy; distance measurement experiments evaluate relative tracking accuracy; and FRE and TRE measurement experiments evaluate point-based image registration performance. The following sections describe each experimental procedure in detail.

### 3.1. Numerical Simulation Experiments

**Figure 4:**
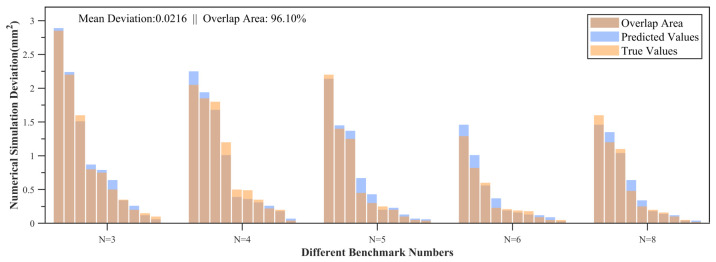
Results of the numerical simulation. The horizontal axis indicates the number of fiducials, and the vertical axis depicts the simulated orientation deviation. Orange columns indicate the actual error values, blue columns indicate the theoretically predicted values, and the khaki area denotes their overlap. Ten simulations were conducted for each number of fiducials, with error values presented in descending order.

To verify the theoretical accuracy of the expected value formula for orientation deviation, a Monte Carlo simulation experiment was conducted. Since the position deviation index is independent of the fiducial point distribution, the experiment focused on the orientation deviation index. The number of fiducial points was set to N∈{3,4,5,6,8}. For each *N*, 10 planar configurations were randomly generated within a 200 × 200 × 200 mm^3^ volume. Gaussian noise following N(0,0.3) was added to the *x*, *y*, and *z* coordinates of each fiducial point. The perturbed positions were then aligned to the initial configuration, and the true orientation deviation index was computed for each simulation. For the same fiducial point distribution, Equation (19) was used to calculate the theoretical expected value. The accuracy of the expected value expression was evaluated by comparing the simulated true deviation values with the corresponding theoretical predictions. The simulation results are shown in Figure 4.

**Figure 5:**
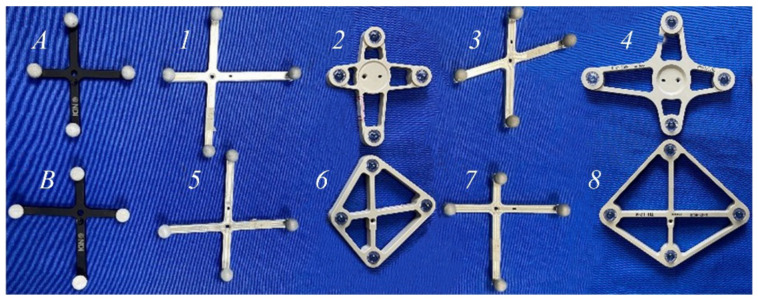
Design parameters were set as follows: 52 mm and 110 mm for the minimum and maximum fiducial segment lengths, 5 mm for the minimum segment length difference, 2° for the minimum segment angle difference, and 1 mm for the iteration step. To minimize processing interference, DRFs were fabricated with an aluminum alloy body and reflective beads (numbers 1, 3, 5, and 7), and a plastic body with reflective lenses (numbers 2, 4, 6, and 8). DRFs A and B correspond to two commercially available DRFs from NDI. All DRFs were geometrically calibrated using NDI 6D Architect software, successfully generating navigation system tool definition files (ROM files) to satisfy subsequent verification requirements.

The experimental results confirmed that the theoretical prediction of the orientation deviation index exhibited a strong positive correlation with the actual error, with an average relative prediction error of less than 5%, demonstrating the formula’s strong predictive capability. As illustrated in Figure 4, the sensitivity of different configurations to error varied significantly, and as the number of fiducial points *N* increased, this influence tended to diminish, aligning with the theoretical derivation. Although increasing the number of fiducial points can theoretically enhance accuracy and occlusion robustness, it also introduces challenges of higher complexity (the number of line segments S=N(N−1)/2 increases sharply) and reduced computational efficiency [1]. Balancing accuracy and real-time performance, N=4 was selected as the number of fiducial points for the subsequent verification experiment. Commonly used design parameters in surgical navigation were adopted, and the candidate tool library was generated using Algorithm 1. The optimal initial configuration was determined according to expected value ranking, after which the compatibility judgment algorithm was applied to select the top eight configurations for processing, as illustrated in Figure 5. In the compatibility test conducted in a multi-tool collaborative working environment, all DRFs in the tool group were successfully validated.

### 3.2. Tip Calibration Experiment

**Figure 6:**
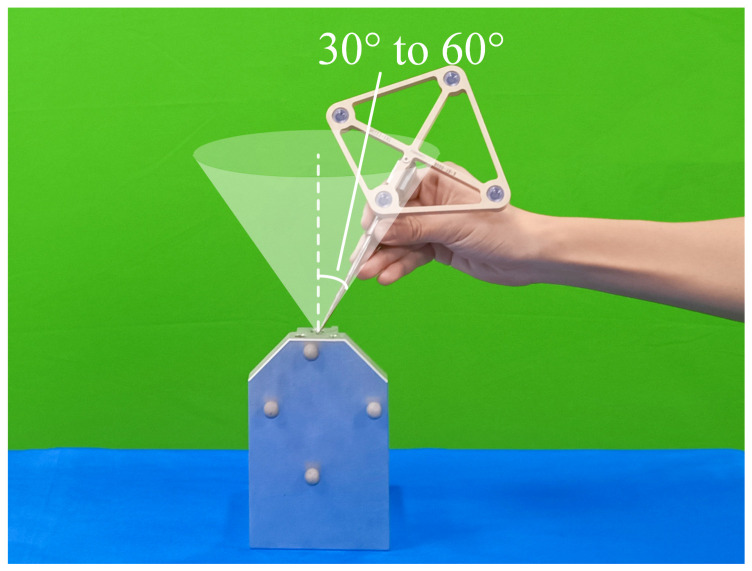
Tip calibration experiment. The DRF is rigidly mounted to the probe via a central aperture. During calibration, the probe tip is held stationary at the center of rotation within the OTS, while the probe–DRF assembly is slowly rotated through angles between 30° and 60°. Throughout each test, the DRF orientation, the probe tip position relative to the DRF coordinate system origin, and the fixed position of the rotation center are maintained.

**Figure 7:**
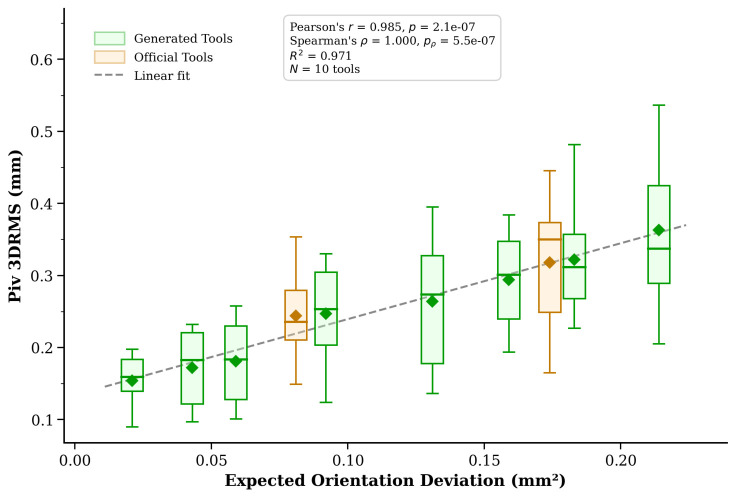
Tip calibration experiment results. The horizontal axis represents the expected orientation deviation for the corresponding configuration, and the vertical axis represents the actual measured tip positioning error. Green boxes represent custom DRFs generated by the algorithm; orange boxes represent commercially available DRFs from NDI. The dashed line shows the linear regression fit ($R^{2}=0.970$). Pearson’s r=0.985 ($p=2.1\times 10^{-7}$), Spearman’s ρ=1.000 ($p=5.5\times 10^{-7}$, exact permutation test).

Tracking the probe tip position is a fundamental function of the OTS, and its calibration accuracy directly affects the probe’s performance. As shown in Figure 6, tip calibration is performed using the pivot calibration method, wherein the rotation matrix $R_{t}$ and translation vector $t_{t}$ of the DRF coordinate system relative to the world coordinate system are continuously collected at *t* time points. The collected data are used with the least squares method to solve for the probe tip position coordinate $X={\left(x,y,z\right)}^{T}$ in the DRF coordinate system. Each DRF calibration was independently repeated 15 times to obtain the results. The final calibration parameters are applied to each time point in the calibration process. The Root Mean Square Error (RMSE), expressed as

(29) $$\mathrm{RMSE}=\sqrt{\frac{\sum_{i=1}^{t}{\left(X_{i}-\bar{X}\right)}^{2}}{t}},$$

is calculated for all position coordinates and used as the accuracy indicator for the tip calibration experiment of each configuration. The tip calibration error distribution is illustrated in Figure 7.

Figure 7 shows the distribution of tip calibration errors for all DRFs. Experimental results indicate a strong positive correlation between the calibration errors and the expected values of the pose deviations (combined N = 10 tools: Pearson’s r=0.985, $p=2.1\times 10^{-7}$; Spearman’s ρ=1.000, $p=5.5\times 10^{-7}$, exact permutation test). Despite differences in manufacturing processes, the accuracy of aluminum alloy/reflective ball DRFs and plastic/lens DRFs is similar. This suggests that the manufacturing method has little impact on the predicted ranking. The actual calibration accuracy of some generated DRFs (numbers 1–3) is superior to that of commercial DRFs. The performance of the self-designed DRFs (numbers 4–6) falls within the range of commercial DRFs. Although the errors of DRFs (numbers 7–8) are slightly higher than those of commercial DRFs, the difference in accuracy is comparatively minor. This is expected, as Algorithm 2 selects compatible tools in order of decreasing theoretical accuracy; configurations selected later in the ranking necessarily have lower predicted accuracy than those selected earlier. Some fluctuations in the repeated tip calibration measurements can be attributed to manual rotation variability and positioning system noise, although the error ranking trend remained unchanged.

### 3.3. Distance Measurement Experiment

**Figure 8:**
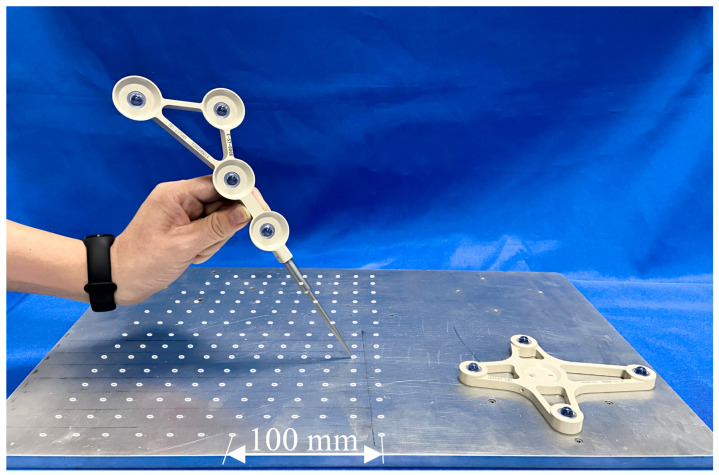
Distance measurement experiment. A high-precision CNC-machined grid plate and DRF are rigidly fixed to the experimental platform. The grid plate’s machining error is ≤0.02 mm, far below the expected optical positioning error. An array of 11×14 groups of precision positioning holes is distributed on the surface, with a spacing of 20 mm between adjacent holes (covering a range of 20–280 mm). An OTS probe (#8700340) is used to collect the coordinates of the grid hole centers.

**Figure 9:**
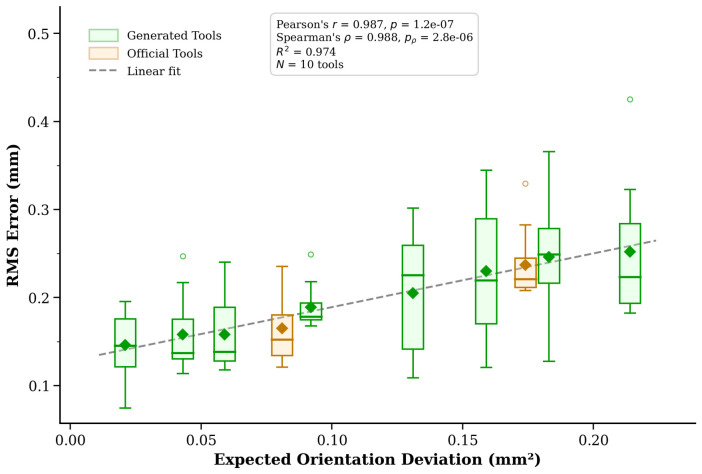
Distance measurement experiment results. The horizontal axis represents the expected orientation deviation for the corresponding configuration, and the vertical axis represents RMSE value of each group of point pairs. Green boxes represent custom DRFs generated by the algorithm; orange boxes represent commercially available DRFs from NDI. The dashed line shows the linear regression fit ($R^{2}=0.974$). Pearson’s r=0.987 ($p=1.2\times 10^{-7}$), Spearman’s ρ=0.988 ($p=2.8\times 10^{-6}$, exact permutation test).

As the true spatial position is difficult to measure directly, the distance measurement error is used as a metric of relative tracking accuracy. As illustrated in Figure 8, the coordinates of the centers of all grid holes in the world coordinate system are measured using a probe, and the real-time pose data of the test DRF is synchronously recorded to enable the conversion of the measured point coordinates to the DRF-fixed coordinate system. Point pairs are selected with a sampling interval of 20 mm along each measurement direction, and 12 groups of point pairs conforming to this interval are selected for measurement. The RMSE value of each group of point pairs is computed to represent the experimental result, while ensuring that the DRF position and the order of experimental points remain the same in each test. The complete experimental process is repeated 15 times for each DRF. The distance measurement experiment results are illustrated in Figure 9, with DRF numbering consistent with the previous experiment.

Figure 9 shows the distribution of measurement errors for all DRFs. Experimental results indicate a clear positive correlation between the errors and the expected values of orientation deviations (combined N = 10: Pearson’s r=0.987, $p=1.2\times 10^{-7}$; Spearman’s ρ=0.988, $p=2.8\times 10^{-6}$, exact permutation test). The measurement errors of two DRFs (numbers 2–3) are identical, which may be attributed to comparable expected values of orientation deviations and limited measurement accuracy. The actual errors of some generated DRFs (numbers 1–3) are lower than those of commercial DRFs. The performance of DRFs (numbers 4–6) falls within the commercial range, whereas the errors of DRFs (numbers 7–8) are slightly higher. Some configurations exhibit outlier measurements, possibly due to the restricted optimal measurement volume of the OTS and the large span of the experimental grid plate. Additionally, ambient light interference during long-distance measurements [26] may reduce the sensitivity of the measurements to differences among DRF configurations. Despite these factors, the differences in actual errors among configurations remain acceptable, and the measured ranking largely agrees with theoretical predictions, confirming the general applicability of the expected orientation deviation in spatial-scale assessment.

### 3.4. FRE and TRE Measurement Experiments

**Figure 10:**
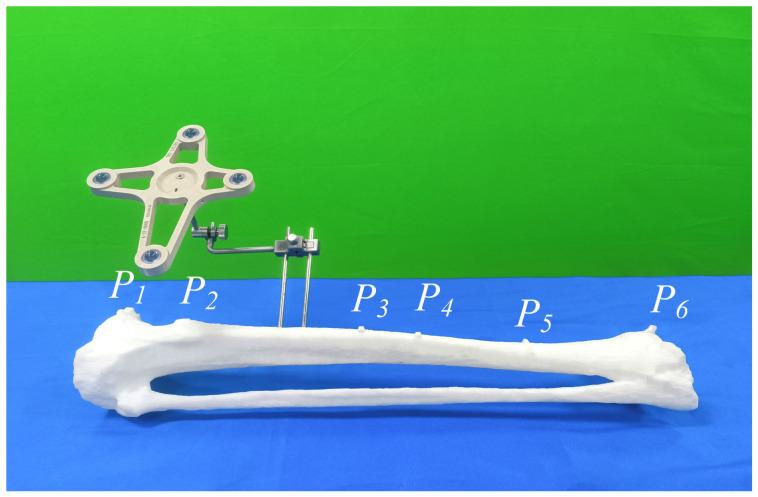
Navigation surgery image registration verification experiment. The DRF to be measured was connected to the fracture model using a dedicated fixture to prevent relative displacement during the experiment. A commercial probe (model #8700340) was used to measure the spatial coordinates of the fiducial points on the fracture model surface. P_1_–P_6_ indicate the six pre-defined landmarks.

The effectiveness of optical tracking tools is often evaluated through point-based registration experiments. The FRE and TRE obtained from the registration results can effectively reflect the performance of the DRF in actual surgical scenarios. The SVD rigid registration algorithm was used, as illustrated in Figure 10. On a 3D fracture model with six pre-defined landmarks, five points were randomly selected as fiducial points, and the remaining point was used as the target point for TRE calculation. Ten replicates were performed on each DRF model, resulting in 20 sets of FRE and TRE data. In each experiment, the spatial coordinates of the fiducials on the fracture model surface were collected and transformed into the DRF coordinate system via a coordinate transformation, resulting in a 3D coordinate set $x_{i}^{w}$ (i=1…6) of the landmarks. Simultaneously, the image coordinates of the corresponding landmarks were manually collected using Mimics software, forming the corresponding point set $x_{i}^{p}$ (i=1…6). Subsequently, point-based rigid registration of $x_{i}^{w}$ and $x_{i}^{p}$ was performed using the first five landmarks, resulting in the registration transformation T. The registration error was then calculated according to Equation (30):

(30) $$FRE=\sqrt{\frac{1}{5}\sum_{i=1}^{5}{\left|x_{i}^{p}-Tx_{i}^{w}\right|}^{2}},\;TRE=\left|x_{6}^{p}-Tx_{6}^{w}\right|$$

**Figure 11:**
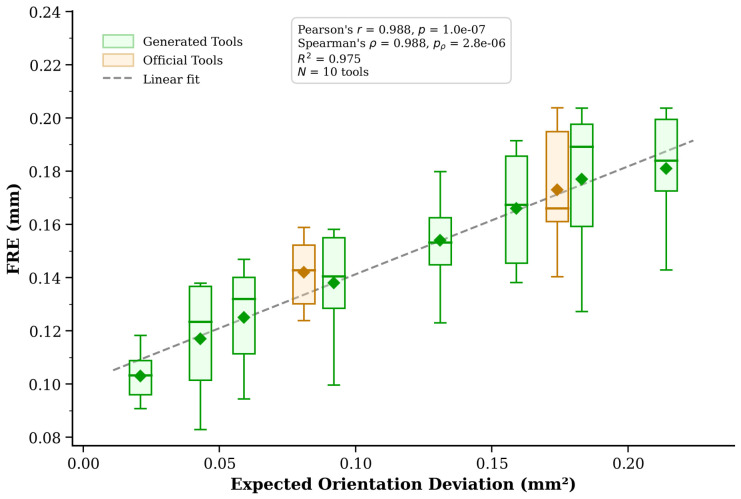
FRE measurement experimental results. The horizontal axis represents the expected orientation deviation for the corresponding configuration, and the vertical axis represents the FRE error. Green boxes represent custom DRFs generated by the algorithm; orange boxes represent commercially available DRFs from NDI. The dashed line shows the linear regression fit ($R^{2}=0.975$). Pearson’s r=0.988 ($p=1.0\times 10^{-7}$), Spearman’s ρ=0.988 ($p=2.8\times 10^{-6}$, exact permutation test).

**Figure 12:**
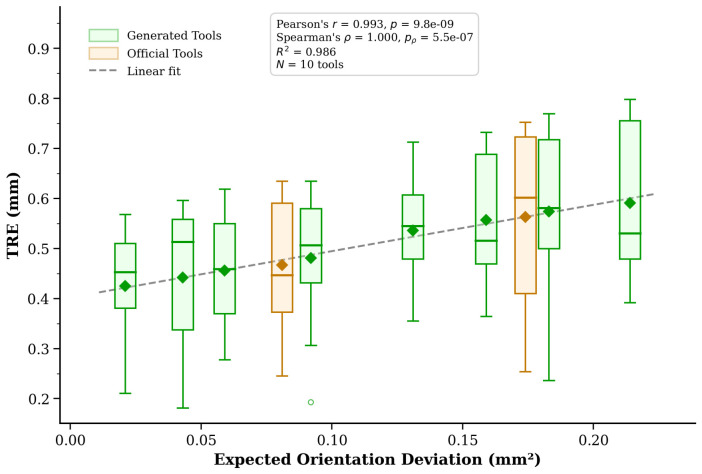
TRE measurement experimental results. The horizontal axis represents the expected orientation deviation for the corresponding configuration, and the vertical axis represents the TRE error. Green boxes represent custom DRFs generated by the algorithm; orange boxes represent commercially available DRFs from NDI. The dashed line shows the linear regression fit ($R^{2}=0.986$). Pearson’s r=0.993 ($p=9.8\times 10^{-9}$), Spearman’s ρ=1.000 ($p=5.5\times 10^{-7}$, exact permutation test).

The smaller the registration error, the higher the availability of the DRF in space. The FRE and TRE results are illustrated in Figure 11 and Figure 12, which show the error mean and maximum/minimum range of each DRF.

Figure 11 shows the FRE measurement values of the generated DRFs (combined N = 10: Pearson’s r=0.988, $p=1.0\times 10^{-7}$; Spearman’s ρ=0.988, $p=2.8\times 10^{-6}$, exact permutation test). The values of DRFs (numbers 1–4) occupy the high-performance range, those of DRFs (numbers 5–6) fall within the commercial DRF range, and the values of DRFs (numbers 7–8) exceed the commercial range. Although the measured FRE ranking generally agrees with theoretical predictions, the commercial DRF shows a slightly higher value than the self-designed DRF (number 5), with a measured difference of 0.02 mm, constituting an exception. This discrepancy may be attributed to different configuration generation strategies or initial calibration conditions affecting the calculation of orientation parameters.

Figure 12 presents the TRE experimental results (combined N = 10: Pearson’s r=0.993, $p=9.8\times 10^{-9}$; Spearman’s ρ=1.000, $p=5.5\times 10^{-7}$, exact permutation test). The TRE error rankings of both generated and commercial DRFs show a strong positive correlation with theoretical predictions. Notably, repeated TRE measurements exhibit greater dispersion than FRE and tip calibration experiments. This is likely due to the higher complexity of TRE measurements, which are affected by additional factors such as spatial registration system errors, leading to inherently greater variability [27]. Despite this, the fluctuation range within each DRF group remains small. Importantly, the trendline of the generated DRF group maintains a good monotonic correlation with predicted values, with overall accuracy slightly exceeding that of the commercial DRF group. Despite inevitable measurement variability, this metric effectively quantifies and differentiates the accuracy levels of DRFs, providing a theoretical basis for selecting high-precision optical tracking tools in clinical applications. These experimental results demonstrate the effectiveness of the expected value of the orientation deviation metric in evaluating the accuracy ranking for both FRE and TRE.

## 4. Discussion

Building on point-based rigid registration theory, this study proposes a unified pose accuracy evaluation metric applicable to both optical probes and DRFs. Through rigorous mathematical derivation, a quantitative relationship is established between this metric, the number of fiducial points, their spatial distribution (moment of inertia along the principal axes), and the fiducial positioning error (Equations (19) and (23) give the expectation and variance, respectively). Theoretical analysis and the derived sensitivity relationships (Equations (26)–(28)) indicate that increasing the number of fiducial points, enlarging their spatial distribution, and reducing the fiducial positioning error all improve the tool’s tracking accuracy, with the variance decreasing faster ($\propto 1/N^{2}$) than the expectation (∝1/N). This conclusion is consistent with previous studies [26] and experimental observations. On this basis, a comprehensive DRF group optimization framework is constructed, comprising exhaustive generation of size configurations, inter-group compatibility assessment, and configuration accuracy ranking and optimization based on the expected value of orientation deviation. This method systematically identifies compatible DRF groups that satisfy both geometric identifiability and accuracy optimization.

A series of validation experiments comprehensively confirmed the effectiveness of the proposed approach. Numerical simulation results show that the proposed metric accurately predicts the theoretical error distribution of various DRF configurations, with prediction errors remaining below 5%. Results from tip calibration, distance measurement, and FRE/TRE experiments consistently demonstrate a strong positive correlation between the measured DRF error and the expected orientation deviation, with Pearson correlation coefficients exceeding 0.98 (all $p<2.2\times 10^{-7}$) and Spearman rank correlations exceeding 0.98 (all $p\leq 2.8\times 10^{-6}$, exact permutation test) across all four independent metrics. Notably, the three custom-designed DRFs with the highest theoretical accuracy achieved the best measured performance in the tip calibration and distance measurement experiments. In the FRE/TRE experiments, most custom-designed DRFs (numbers 1–4) achieved accuracy comparable to or modestly better than the commercial DRFs, demonstrating the capability of the optimization design framework (Algorithms 1 and 2) to generate and select high-precision DRF configurations. Compared with existing methods, the generation strategy in [28] constructs a candidate configuration pool through random sampling and selects six configurations for testing, with a measured FRE of approximately 0.15 mm. In contrast, the algorithm proposed in this study incorporates a precision ranking mechanism to select the top candidate configurations. When the same number of tools (six) are drawn from the ranked library, the proposed algorithm reduces the average FRE to 0.13 mm.

The absolute magnitude of the accuracy improvement over commercial DRFs (0.02–0.05 mm across metrics) should be interpreted within the broader context of surgical navigation error budgets. In procedures such as spinal pedicle screw placement, the clinically acceptable target registration error is typically 1–2 mm [26]. Within this budget, every sub-millimeter improvement at the tracking level increases the safety margin available for other error sources including image-to-patient registration, intraoperative tissue deformation, and surgeon variability. More importantly, however, the primary value of the proposed framework is not the marginal accuracy gain over any specific commercial tool, but rather that it achieves commercial-grade or modestly improved accuracy through an *open, reproducible, and customizable design methodology*—a capability that has historically been accessible only through proprietary industrial optimization. The framework enables three categories of practical benefit: (1) for researchers developing novel navigation algorithms, it provides a systematic method for generating high-accuracy reference tools with known error characteristics; (2) for engineers designing procedure-specific instruments, it offers a principled approach to optimizing tool geometry under application-specific workspace constraints (e.g., sterilization requirements, robotic end-effector mounting, or minimally invasive access corridors); and (3) for the broader surgical navigation community, it makes transparent the design rationale that underlies tracking accuracy, facilitating education, benchmarking, and reproducibility.

The proposed framework also includes a customizable weighted evaluation function. For applications where translational and rotational errors have different clinical significance, the accuracy metric can be generalized as $W=w_{t}\cdot {\langle |\Delta t|}^{2}\rangle +w_{r}\cdot {\langle |\Delta R|}^{2}\rangle$, where the weights $w_{t}$ and $w_{r}$ can be tuned to reflect application-specific requirements. For instance, spinal pedicle screw placement may prioritize angular accuracy ($w_{r}>w_{t}$), while surface-based registration tasks may prioritize positional accuracy ($w_{t}>w_{r}$). The variance expressions provided in Equations (23) and (25) further allow users to assess not only the expected accuracy but also its expected variability for a given configuration.

Nevertheless, several limitations of this study should be explicitly acknowledged. First, the error model assumes that fiducial positioning errors are independent, identically distributed, isotropic, and approximately Gaussian. In complex clinical environments, factors such as partial marker occlusion, directional lighting, and multi-path reflections may introduce correlated or non-Gaussian noise components. The extension of the error model to accommodate such noise structures represents an important direction for future work. Second, the configuration generation algorithm (Algorithm 1) in its current implementation focuses on planar fiducial configurations. While the underlying accuracy metric (Equations (19) and (23)) is general and applies to any spatial point distribution, extending Algorithm 1 to three-dimensional non-coplanar configurations requires a higher-dimensional search space and additional geometric constraints. Third, the proposed error model addresses static pose estimation accuracy; dynamic tracking performance—affected by instrument velocity, OTS sampling rate, and marker motion blur—is not explicitly modeled. In typical surgical scenarios, tracking tools move at moderate speeds relative to the 20–60 Hz sampling frequency of modern OTS, and static accuracy remains the primary design consideration for DRF configurations. Fourth, the compatibility discrimination thresholds $\Delta d_{\min}$ and $\alpha_{\min}$ are currently based on a semi-theoretical analysis; a fully rigorous derivation of optimal thresholds via formal confusion-probability modeling is deferred to future work. Fifth, the current work derives the expectation and variance of the orientation deviation but does not provide analytical worst-case error bounds (e.g., via concentration inequalities); such bounds, which would require additional assumptions about noise tail behavior, represent a direction for future theoretical development. Finally, several experimental design limitations should be noted. The experimenter was not blinded to tool identity during testing, and tool testing order was not randomized; although the automated nature of OTS measurements mitigates potential operator bias, standardized protocols would strengthen future studies. The commercial reference group consists of only two NDI DRFs, which limits the generalizability of the accuracy comparison. Expanding the commercial baseline to a larger and more diverse panel of reference tools would improve external validity. Furthermore, experimental validation was conducted in an ideal, controlled laboratory setting. The influence of clinical factors such as tissue occlusion, instrument manipulation, and varying ambient lighting conditions warrants further investigation in realistic surgical environments.

Regarding the statistical methodology, all Pearson correlation *p*-values were computed using the standard two-tailed *t*-test with N−2 degrees of freedom. Spearman rank correlation *p*-values were computed using exact permutation tests (evaluating all 10! rank permutations) rather than asymptotic approximations, ensuring valid inference for the sample size of N=10 tools. The very large effect sizes observed (Pearson r>0.98, corresponding to $R^{2}>0.97$) provide high statistical power despite the moderate sample size: for r=0.98 and N=10, the power exceeds 0.99 at α=0.001. The consistent replication of this strong correlation across four independent experimental metrics—each with distinct measurement protocols and error sources—further strengthens confidence in the relationship between the theoretical accuracy metric and measured tracking performance.

In summary, this paper makes three principal contributions: (1) an open, reproducible design methodology that formalizes knowledge historically proprietary to commercial tracking system vendors, enabling the systematic design of high-precision optical tracking tools; (2) a constraint-driven automated framework that generates and screens compatible multi-tool configurations while satisfying geometric uniqueness and inter-group compatibility constraints, with theoretical characterization of both the expectation and variance of the accuracy metric; and (3) experimental validation demonstrating that tools designed by this framework achieve accuracy comparable to or modestly exceeding that of commercial references, with statistically significant correlation between the theoretical metric and all measured error indicators (Pearson r>0.98, all $p<2.2\times 10^{-7}$; Spearman ρ≥0.988, all exact $p\leq 2.8\times 10^{-6}$). The open nature of this methodology makes systematic high-precision tool design accessible to the broader surgical navigation research community.

## 5. Conclusions

This paper has presented a constraint-driven automated framework for optimizing multi-tool fiducial configurations in surgical navigation. Beyond the specific numerical results, three broader implications of this work merit emphasis. First, by making the design rationale underlying tracking accuracy transparent and reproducible, the proposed framework lowers the barrier for researchers and engineers who require custom optical tracking tools but lack access to proprietary industrial optimization pipelines. Second, the analytical characterization of both the expectation and variance of the accuracy metric—together with the parameter sensitivity relationships—provides a principled basis for informed design trade-offs (e.g., fiducial count vs. computational complexity, spatial spread vs. workspace constraints). Third, the constraint-driven compatibility screening mechanism formalizes inter-tool discrimination requirements that, to date, have been handled largely through ad hoc empirical testing. Future work will extend the framework to three-dimensional non-coplanar configurations, incorporate dynamic tracking error models and marker occlusion probabilities, derive analytical worst-case error bounds, and validate performance under realistic clinical conditions including tissue occlusion and varying ambient lighting. 

## Data Availability

The data presented in this study are available on request from the corresponding author.
